# A Novel Technique for the *In Vivo* Imaging of Autoimmune Diabetes Development in the Pancreas by Two-Photon Microscopy

**DOI:** 10.1371/journal.pone.0015732

**Published:** 2010-12-23

**Authors:** Ken Coppieters, Marianne M. Martinic, William B. Kiosses, Natalie Amirian, Matthias von Herrath

**Affiliations:** 1 Type 1 Diabetes (T1D) Center at the La Jolla Institute for Allergy and Immunology, La Jolla, California, United States of America; 2 Core Microscopy Facility, The Scripps Research Institute, La Jolla, California, United States of America; University of Bremen, Germany

## Abstract

Type 1 diabetes (T1D) is characterized by the immune-mediated destruction of beta cells in the pancreas. Little is known about the *in vivo* dynamic interactions between T cells and beta cells or the kinetic behavior of other immune cell subsets in the pancreatic islets. Utilizing multiphoton microscopy we have designed a technique that allows for the real-time visualization of diabetogenic T cells and dendritic cells in pancreatic islets in a live animal, including their interplay with beta cells and the vasculature. Using a custom designed stage, the pancreas was surgically exposed under live conditions so that imaging of islets under intact blood pressure and oxygen supply became possible. We demonstrate here that this approach allows for the tracking of diabetogenic leukocytes as well as vascularization phenotype of islets and accumulation of dendritic cells in islets during diabetes pathogenesis. This technique should be useful in mapping crucial kinetic events in T1D pathogenesis and in testing the impact of immune based interventions on T cell migration, extravasation and islet destruction.

## Introduction

In type 1 diabetes (T1D), destruction of beta cells located in the islets of Langerhans throughout the pancreas is extremely difficult to study owing to the organ's inaccessible location, diffuse tissue architecture and abundance of potentially harmful digestive enzymes that make it difficult to obtain biopsy tissue samples[1]. Despite some differences compared to the human pathophysiology, our knowledge of how T1D develops has benefited significantly from studies in rodent models such as the non-obese diabetic mouse (NOD)[2]. In mouse and man, documentation of autoimmune events in the pancreatic islets (a process termed ‘insulitis’) has been traditionally achieved by histological techniques in cross-sectional studies[3]. While such data provide a one-time ‘snapshot’ of islet destruction, there is no clear knowledge of the precise cellular dynamics involved in this process.

Since first reported by Denk and coworkers[4], two-photon microscopy has been applied extensively to image immune cells in intact lymphoid organs[5], [6]. The major advancement associated with the technique is the use of a pulsed infrared laser for fluorescent dye excitation[7]. This high excitation wavelength allows for deep tissue imaging and its low energy constrained to the focal plane limits phototoxicity. As a consequence, two-photon microscopy has become the technique of choice to assess the dynamic behavior of immune cells *in vivo*. To date, however, the handful of studies that applied two-photon microscopy in the context of autoimmune diabetes were limited to the pancreatic draining lymph nodes[8], [9], [10], whereas the situation in the pancreas remains uncharted territory. One group performed 2-photon microscopy on islets transferred into the anterior chamber of the eye, but the immune privileged nature of the site precludes inclusion of the hallmark autoimmune component of T1D[11]. Another recent study by Nyman et al introduced the use of intravital, high-speed confocal scanning in order to determine blood flow dynamics in islets[12]. The latter approach, however, poses considerable constraints in terms of imaging depth and laser cytotoxicity and thus is unsuitable for tracking immune cells around islets over longer time spans.

The dynamic behavior of immune cells is profoundly dependent on physiological conditions[13], and it is particularly questionable whether circulatory deprivation in explanted pancreas preparations would leave the true *in situ* parameters of diabetogenic immune responses unaltered. We report here a novel approach to visualize the kinetic properties of immune cells during the development of diabetes in the intact pancreas and islets of living animals. As such, we provide the first real-time visualization of leukocyte-beta cell interactions and dendritic cell recruitment to the islets.

## Results

### Design of a surgical approach for intravital two-photon studies in the pancreas

**Figure 1:**
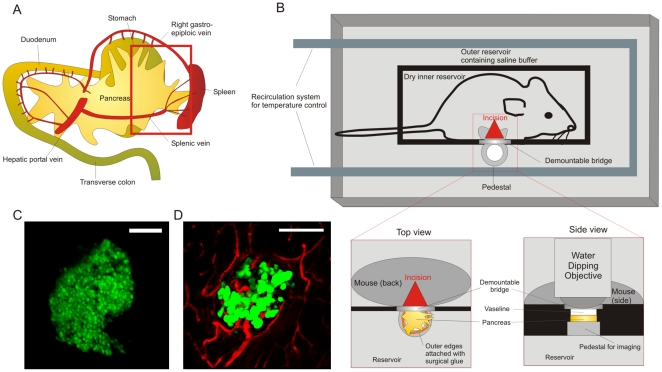
Surgical approach and maintenance of physiologic conditions during intravital imaging of the pancreas. **A**. Schematic overview of the anatomical orientation of the pancreas in rodents. In order to gain access to the pancreatic tail region, we cauterized the splenic vessels and performed a splenectomy. The red rectangle indicates the region of the pancreas subjected to imaging. **B**. Design of a custom-made imaging reservoir for two-photon imaging of the pancreas. The outer reservoir is filled with saline buffer, covers the organ and is continuously maintained between 36.5 and 37 degrees Celcius by a recirculation system. The inner reservoir containing the animal is sealed from the buffer by a barrier (black) and a demountable bridge at the site of the imaging pedestal. To achieve sealing without pressuring the tissue, Vaseline is applied between bridge and pancreas. The pancreatic tail region is gently exposed and its outer edges attached to the pedestal with surgical glue. Finally, the water dipping objective is lowered onto the pancreas for imaging. **C**. Typical *in vivo* appearance of a healthy islet, showing coherent beta cell architecture and uniform GFP expression (MIP of 76 z-planes spaced 2 µm; pixel w/h: 1.2 µm; corresponds to Movie S1). **D**. Islet microvasculature (red) as revealed by intravital imaging after injection of Texas-Red labeled dextran. (MIP of 43 z-planes spaced 5 µm; pixel w/h: 0.6 µm). Scale bars in C and D are 100 µm.

The anatomical location of the pancreas, in between the curve of the duodenum (widest part; ‘head’) and the spleen (smallest part; ‘tail’), calls for a careful approach to avoid potentially fatal organ damage or bleeding during imaging (Figure 1A). In addition, the pancreas is softer and less compactly organized than most other organs, requiring avoidance of excessive force during exposition. We chose to seek access from the splenic side and image the tail region after cauterization of the splenic arteries. This leaves the vascular supply originating from the superior and inferior pancreaticoduodenal arteries intact. Successful intravital two-photon imaging is dependent on 3 major factors: (1) immobilization of the tissue to minimize movement artifacts; (2) immersion in heated buffer at precise physiological temperature for use with a water dipping objective; (3) proper maintenance of anesthesia and body temperature of the animal. We built a custom-designed reservoir and incorporated a heating supply to satisfy these requirements (Figure 1B). In this way, the tail region of the pancreas could be gently exposed and its outer edges glued to an imaging pedestal. The surrounding buffer was maintained at 37°C by a recirculation system, which has the combined benefit that the animal's body temperature is preserved. Importantly, the animal's abdominal area itself was not exposed to the buffer, as water influx was prevented by a small plastic barrier sealed with Vaseline in between the inner reservoir and the imaging pedestal. Applying Vaseline ensures proper sealing while avoiding any pressure on the tissue that may block circulation. The minor remaining breathing artifact was corrected post acquisition by use of the ImageJ registration plugins TurboReg and StackReg[14]. Typical *in vivo* 2D islet morphology as imaged by two-photon microscopy is depicted in Figure 1C and as a 3D rotating rendering in Movie S1. This intravital approach has the notable advantage of allowing for detailed visualization of pancreatic islet vasculature, as is achieved by intravenous injection of fluorescently labeled agents (Figure 1D; Movie S2). Moreover, the intact structural integrity of the local microvessels clearly indicates that sufficient blood supply is maintained around the islet site and adds physiological significance to our findings.

### Determining leukocyte motility and interactions with beta cells in the pancreas

**Figure 2:**
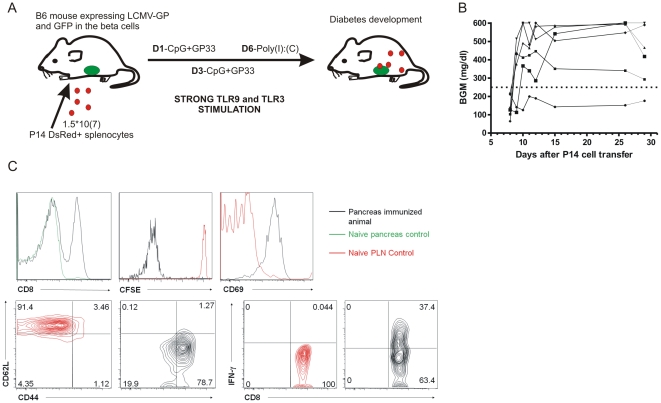
An acute diabetes model suitable for *in vivo* pancreas imaging. **A**. Genetically labeled GP33-specific (‘P14’) splenocytes were adoptively transferred into fluorescent reporter mice harboring green beta cells that express LCMV-GP antigen. The cells were stimulated *in vivo* by repeated stimulation with peptide, CpG and Poly-IC (TLR ligation). **B**. These mice develop highly synchronized autoimmune diabetes with clinical onset between day 9–10. BGM: blood glucose measurement **C**. Pancreatic draining lymph nodes (PLN) and pancreas (PAN) were harvested at time of onset (day 8 post transfer) and stained for the P14 TCR chains Vá2 and Vâ8.1/2, CD8á, CD69, CD44 and CD62L. In conjunction with CFSE dilution and ICCS for IFN-ã, this analysis reveals the influx of highly activated, memory phenotype GP33-specific CD8 T cells around the time of onset. PLN cells from naïve animals were used as controls for comparison since none of the cells homed to the pancreas in the absence of stimulation.

We have previously established an antigen-specific, virus-free model of beta cell destruction that relies on adoptive transfer of CD8 T cells ([15] and manuscript in press[16]). In brief, breaking tolerance to enable these cells to access the islets requires a remarkably extensive stimulation protocol, mimicking some of the effects of acute viral infection by repeated stimulation, immunization and TLR ligation (Figure 2A). As a result, recipient mice develop diabetes in a highly synchronized fashion 9–10 days after transfer (Figure 2B). Prior to *in vivo* microscopy, we characterized the phenotype of the T cells within the transferred population at diabetes onset in the pancreas (Figure 2C). Transferred cells that migrated into the pancreas had vigorously proliferated, acquired an effector memory phenotype, and were producing considerable amounts of IFN-ã. Our intent was to enhance our chances of detecting transferred cells around the limited population of islets accessible by two-photon microscopy through the acute and synchronized nature of autoimmune diabetes observed in this model. From earlier studies we concluded that the optimal time window for imaging of T cells and beta cells was on day 7–8, right before onset of clinical diabetes.

**Figure 3:**
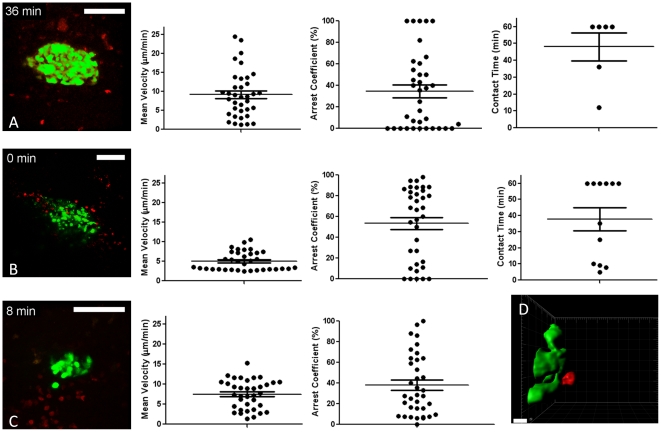
*In vivo* imaging captures T cell motility and interactions with beta cells in the pancreas. **A**. Corresponds to *left panel* of Movie S3 (MIP from 15 z planes, 11 µm apart, pixel w/h: 0.6 µm). Mean track velocities, arrest coefficients and beta cell contact times were determined after 3D tracking. **B**. As immune-mediated destruction advances, the GFP signal disintegrates in what appear to be multiple small GFP-containing vesicles (MIP from 15 z planes, 12 µm apart, pixel w/h: 0.9 µm; corresponds to *middle panel* of Movie S3). Mean track velocities, arrest coefficients and beta cell contact times were determined after 3D tracking. **C**. Reduced islet size and considerable infiltrate is observed (MIP from 15 z planes, 7.2 µm apart, pixel w/h: 0.5 µm; corresponds to *right panel* of Movie S3). Only mean track velocities and arrest coefficients were determined as only 1 cell was found to establish prolonged contact with beta cells. **D**. Isosurface rendition of a T cell (red) in contact with beta cells (green), derived from Movie S4 (*left panel*). White scale bars are 100 µm, except for D, 10 µm.

We applied the peptide/adjuvant-induced model to capture the invasion process within islets at various stages of destruction (Figure 3; Movie S3). For these studies, we crossed the CD8-TcR transgenic P14 strain to mice ubiquitously expressing the red fluorescent protein DsRed[17]. This setup allowed us to track ‘red’ CD8 T cells to attack ‘green’ islets and achieve sufficient spatial resolution to characterize CD8 T cell-beta cell contacts.

Especially in Figure 3B, the coherent GFP signal as observed under baseline conditions (see Figure 1C) is fragmented over what appear to be numerous small apoptotic vesicles. In these proof-of-concept studies we transferred unpurified splenocytes in order to replicate the high diabetes incidence as presented in Figure 2B. Considering that P14 mice typically harbor only ∼2% CD4 T cells in their splenocyte population, we therefore assume that the majority of visualized events were CD8 T cells and that B cells were the second most frequent population during the late stages of insulitis, similar to what is observed in human islet pathology[18].

After image registration in order to eliminate the breathing artifact, three-dimensional tracking was performed on the time series depicted in Movie S3 (Figure 3A–C). Calculation of mean track velocities revealed variable cellular speeds, and most velocities are broadly within the range observed previously by tracking of T cells in the lymph nodes[5]. Interestingly, cellular velocities were markedly lower (*P* = 0.0021 versus 3A; *P* = 0.0038 versus 3C) around the islet that showed incoherent GFP signal (3B). Particularly in this movie S3B, cellular arrest in the proximity of beta cells became prevalent (Figure 3D; Movie S4) as evidenced by the high arrest coefficients (Figure 3B; *P* = 0.0328 versus 3A and *P* = 0.0834 versus 3C). However, we found that only a minor fraction of the cells established prolonged interactions with the beta cells, but in these cases a stable interaction could last for more than an hour (Figure 3A,B). In general, stable contacts with beta cells were rare, as for instance only 1 cell was found to establish prolonged interaction in movie S3C.

Given the extensive amount of inflammation observed in Movie S3 (particularly *right panel*), we expected to see some of the beta cells become GFP negative over time as they would commit to apoptosis. However, beta cell apoptosis was only rarely detected during our imaging time frame of 1–4 hours, despite the high numbers of diabetogenic CD8 T cells present and further studies are underway to precisely quantify such events.

We conclude that our approach enables us to efficiently detect the kinetics of autoreactive immune cells in the proximity of pancreatic islets during diabetes development.

### Imaging dendritic cells under steady-state versus insulitic conditions

**Figure 4:**
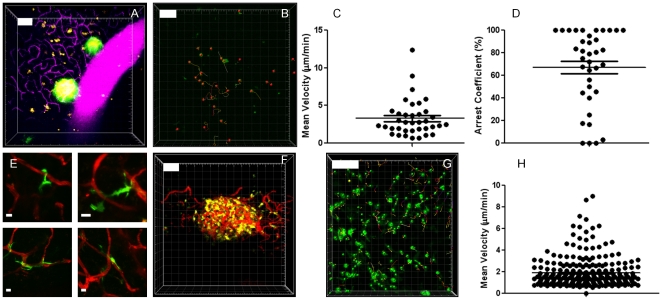
Pancreatic dendritic cells constitute a static population in the steady state, but are recruited to the islets under conditions of inflammation. **A**. Dendritic cells (yellow) can be seen in the normal pancreas at relatively low densities, in no particular association with the islets (MIP of 35 slices spaced 8 µm apart; pixel w/h: 1.5 µm; corresponds to Movie S5, *left panel*). Injection of a Texas-Red labeled dextran reveals the dense vascular network (magenta) in the pancreas. Yellowish color of the islets is due to spectral bleedthrough. **B,C,D**. Tracking of DC in the exocrine pancreas under normal conditions was performed in triplicate (representative experiment in **B**) and shows low average track velocities (**C**) and a high portion of cells that virtually arrested during imaging as judged from their arrest coefficients (**D**). **E**. Dendritic cells remain in close interaction with the local microvasculature under normal conditions. Images are MIPs spanning total z-volumes of 50 to 70 µm with a step size of 3 to 5 µm. Pixel w/h: 0.3 µm (lower planes), 0.2 and 0.1 µm (upper left and right, resp.). **F**. Upon diabetes induction, DC accumulate (MIP of 30 planes spaced 6 µm apart; pixel w/h: 1.5 µm; corresponds to Movie S6, *middle panel*) and completely arrest for prolonged periods of time in dense clusters, wedged in an around the islets. Tracking of CD11c+ cells in the exocrine pancreas showed little motility (**G, H**) Experiments were performed in triplicate (see 3 panels in Movie S6). The vasculature can be seen in red. White scale bars are 100 µm, except E, 10 µm. All tracking data are representative of duplicate experiments.

The CD11c-eYFP reporter mouse represents a valuable tool for the efficient detection of endogenous dendritic cells by two-photon microscopy[19]. Under baseline conditions, dendritic cells in the pancreas are predominantly slowly moving cells (Figure 4B,C; Movie S5) and, except for ∼10 intra-islet DC's[20], are not significantly associated with the islets (Figure 4A; Movie S5). The cells spent a significant portion of their time in virtual arrest (Figure 4B, D). In line with a report by Calderon et al, we find that dendritic cells maintain intimate contacts with the local microvasculature[20] (Figure 4E, Movie S5 *right panel*). The constant extension/retraction of processes could be seen, characteristic of their constant environmental sampling.

Upon induction of diabetes, however, dendritic cells were found to accumulate in dense clusters around the islets, wedging in between the remaining beta cells (Figure 4F; Movie S6). Dendritic cells were seen to be gradually recruited to the site of inflammation (Movie S6, arrows in *left panel*). Under these conditions, dendritic cells virtually arrest at the site of inflammation (Figure 4G,H).

## Discussion

We have presented here an *in vivo* approach to visualize the cellular kinetics of diabetes development in a novel adoptive transfer model based on the well-studied viral RIP-LCMV model. In a 2008 review article, we previously theorized an approach for ‘real-time imaging of the pancreas during development of diabetes’, based on our experience with an inverted confocal microscope[15]. Upon implementation, we found here that the diabetes transfer model proposed in that article is indeed suitable for imaging purposes but its rudimentary surgical approach was soon abandoned.

Taking into account the notorious inaccessibility of the pancreas for *in vivo* studies we have taken advantage of two-photon microscopy for deep-tissue intravital imaging of cellular interactions in an intact soft organ. In doing so, we provide a first impression of the dynamic interplay between diabetogenic immune cells and beta cells.

### Advantages

First, we developed a surgical procedure which allows us to expose the pancreatic tail region to the extent that it could be placed under our upright microscope objective. We estimate that this technique enables us to access approximately one third of the pancreas from the tail side. Any islet in that region, regardless of its size, can be reliably visualized, provided it lies close enough to the surface (∼250 µm two-photon depth limit).

We next built a custom-designed imaging reservoir enabling proper maintenance of hydration and tight temperature control. To date, Nyman et al were the only group to achieve *in vivo* imaging of pancreatic beta cells at cellular resolution[12]. Our present method not only offers improved physiological control but, in conjunction with adoptive transfer of fluorescently labeled effector cells, allows for visualization of diabetogenic responses *in situ*. Like in the work by Nyman et al, microvascular blood flow is maintained around the islets in our studies and accentuates the validity of our intravital approach with respect to obtaining physiologic, meaningful results.

An acute transfer model for autoimmune diabetes was employed by introduction of fluorescently labeled splenocytes harboring diabetogenic TCR-transgenic CD8 T cells into recipients expressing both antigen and GFP in their beta cells. We propose that two factors are pivotal for successful two-photon imaging of islet autoimmunity: induction of synchronized insulitis and genetic labeling of the transferred cell population with a strong fluorophore. The first requirement is associated with the limited amount of islets that are close enough to the surface to be scanned by common two-photon microscopy systems. Synchronicity of beta cell decay by use of an aggressive, acute-onset induction protocol therefore enhances the chances of capturing beta cell destruction in real-time. In conjunction with the availability of mice expressing GFP under the insulin promoter in both B6 and NOD background and strains expressing red or far-red fluorophores controlled by a variety of promoters, we believe there is ample opportunity to translate our approach to other diabetes models. In the NOD mouse, several options exist to synchronize/accelerate insulitis such as injection of cyclophosphamide[21], adoptive transfer of highly diabetogenic T cell clones[22] or even viral infection with coxsackievirus[23].

### Limitations

In advancing two-photon microscopy towards *in vivo* imaging of the pancreas, we have encountered some limitations that are inherent to deep-tissue imaging. The micrometer resolution in conjunction with the high number of optical stacks required for reconstruction of an islet over time introduces a considerable breathing artifact. We successfully corrected for this using commonly available software, avoiding image distortion or affecting the results of cellular tracking.

In the current study, we aimed to capture ongoing insulitis within entire islets. The z-step size we used to acquire such large volumes is higher than the conventionally used five microns[24], [25] and likely introduces some degree of inaccuracy in determining the center of cell mass during 3D tracking. In particular some broadening can be expected in the velocity distribution[26]. That said, smaller z-stepping is certainly possible for more precise measurements of e.g. instantaneous velocity of individual cells in the islets, but will come at the expense of captured cell numbers, which in our case are scattered around the pancreatic islet.

Since the imaging depth is in the order of a few hundred µm, only the most superficial subset of islets is accessible. It is conceivable that this restraint leads to some degree of experimental bias as the largest islets are predominantly located in the core of the pancreas. On the other hand, it was shown previously that the smallest, peripherally located islets are the first to be destroyed during diabetes development in the NOD model[27]. This population may therefore in the NOD mouse constitute an ideal target for imaging T cell behavior at the stage of early immune-mediated beta cell loss.

This technique is to be considered terminal and is therefore not intended for extended longitudinal studies of beta cell mass fluctuation. The imaging time is in the order of hours and is thus relatively short in comparison to body window-enabled methods [28]. Techniques such as *in vivo* optical coherence microscopy[29] or ex vivo optical projection tomography[27] are more suitable for the purpose of following beta cell mass over time but are currently restricted to imaging at islet resolution. Instead, the technique presented here is designed to image individual T cell interactions with beta cells *in vivo* over the course of hours in order to assess parameters such as their retention time in islets, potential migration between islets or interactions with the vasculature.

### Interpretation

Although we wish to avoid overinterpretation of the presented dataset, we observed a low overall frequency of prolonged leukocyte-beta cell interactions. This is consistent with our earlier notion that most beta cells in this model die by cytokine-mediated effects rather than through perforin[30]. The observation that a small fraction of the cells does arrest in contact with beta cells for extended time periods is reminiscent of the results obtained in tumor models, where a recent study suggested that CTL establish interactions with tumor cells lasting an average of 6 hours [25]. However, it remains uncertain whether these stable contacts represent beta cell killing or rather antigen recognition, although the highly activated memory phenotype of the infiltrating population may suggest the former event. Our studies also suggest that beta cell death requires the presence of considerable numbers of activated CD8 T cells patrolling in the immediate vicinity of the islet site. The fact that actual beta cell death was only rarely observed is in line with our data in the conventional RIP-LCMV.GP model (manuscript in preparation). This may in part explain why T1D development in humans requires several years, particularly when the substantially lower degree of insulitis is taken in consideration as compared to mouse models [18]. Additionally, there likely is a delay between apoptosis commitment and loss of GFP signal, which may lead to an underestimation of ‘dying’ cell numbers at any given time.

### Conclusion

In summary, we have developed a novel approach for live *in vivo* assessment of diabetogenic immune responses in the pancreas and islets of Langerhans under physiological conditions. We believe this will greatly enhance our understanding of the kinetic events underlying beta cell destruction and its counter-regulation by suitable immune-based therapies.

## Methods

### Mice

We crossed B6.Cg-Tg(CAG-DsRed*MST)1Nagy/J mice[17] mice to P14 mice[31], which express a TCR specific for the GP33 CD8 epitope of the LCM virus. Mice expressing GP antigen from the LCMV-WE [32] strain under control of the rat insulin promoter (RIP-GP) were bred to a strain with beta cell-restricted GFP expression (MIP-GFP)[33]. For visualization of dendritic cells, mice expressing eYFP under the control of the CD11c promoter were used[19] and bred to the MIP-GFP/RIP-GP strain. All animal procedures were approved by the Animal Care Committee of the La Jolla Institute for Allergy and Immunology (protocol nr. AP121-MvH3-0510).

### Diabetes induction protocol

On day 0, a single cell suspension of 1.5*10(7) P14 splenocytes were transferred into recipient mice via retroorbital injection. On day 1 and 3, mice were intraperitoneally injected with 2 mg of GP33 peptide (Abgent, NH2-KAVYNFATM-COOH) in ultrapure water containing 50 ug of CpG adjuvant (Sigma). Finally, on day 6, mice received 500 ug of polyinosinic:polycytidylic acid (Poly I:C) i.p. (Sigma). All imaging was done on day 7 and 8.

### Flow cytometry

Single cell suspensions were prepared from spleen, pancreatic draining lymph nodes and pancreas. Red blood cell lysis was performed on splenocyte preparations. All antibodies used were purchased from BD. For intracellular cytokine staining, cells were restimulated with GP33 antigen for 5 hours in the presence of Brefeldin A (GolgiPlug, BD), stained for surface markers and subsequently fixed and permeabilized (Cytofix/Cytoperm, BD) for intracellular staining. Acquisition was performed on an LSRII flow cytometer (BD).

### Surgery and temperature control

Mice were injected with an initial dose of 90 mg/kg ketamine hydrochloride and 15 mg/kg xylazine hydrochloride, which we previously titrated for efficient and safe induction of long-term anesthesia. Mice were given half this dose approximately every 45 minutes. The abdominal area was shaved and a longitudinal incision was made at the site of the spleen. The spleen was gently exposed with the pancreatic tail region attached and the splenic vessels were cauterized and splenectomy was performed. The edges of the pancreatic tail region were attached with surgical glue (Histoacryl, Braun) to the pedestal of a custom-made imaging reservoir. The imaging reservoir's temperature was controlled by a recirculation system based on a Gaymar T/Pump. Local temperature at the pedestal was checked with a sensitive probe (Warner instruments). Heart rate and arterial oxygen saturation were monitored using a MouseOx pulse oximeter (Starr Life Sciences Corp) and were found to be similar in animals undergoing surgery as compared to anesthetized controls.

### Two-photon imaging and analysis

We used a Leica SP5 microscope system equipped with an IR Laser Chameleon ultra ps, tunable from 690 to 1040 nm. For this study we used 3 channels with non-descanned detectors with filter sets suitable for separation of GFP (513/17 nm), eYFP (542/27) and DsRED (692/40 nm) in combination with 520 nm and 560 nm dichroic mirrors. We used an Olympus Super 20 (XLUMPLFL20XW, NA0.95) water dipping objective controlled by a piezo Z-focus element (Piezosystem Jena). At 900 nm excitation wavelength, photon penetration and efficient detection is up to 250 µm deep into the tissue. Laser power at the sample was 220 mW and was determined using a Thorlabs PM100D power meter after galvo-scanning was stopped (thus parking the beam) and AOTF blanking was shut off. Post-acquisition enhancement (i.e. ‘normalization’ or altering the range of pixel intensity values for viewing purposes), MIP and panel combination were performed using ImageJ. The Turboreg and Stackreg plugins[34] were used for registration within Z-stacks (i.e. elimination of breathing artifact). 3D tracking was performed using Imaris (Bitplane) after correction for tissue drift. True cellular contact was confirmed by looking at the individual Z-planes. In most movies, spectral overlap at the islet site was corrected for by subtracting signal from the ‘red’ from the ‘green’ channel, rendering all islets uniformly green for the sake of clarity. Finally, image sequences were converted to TIFF or AVI format. Pixel width and height (w/h) are provided in microns for each image and movie in the legends.

The confinement ratio corresponds to the ratio of the distance between the initial and the final positions of each cell to the total distance covered by the same cell. The arrest coefficient is defined as the proportion of time each cell's instantaneous velocity (calculated for every time interval) is <3 µm/min.

Visualization of vasculature was achieved by the i.v. injection of 250 µg of 70 kDa Texas-Red labeled dextran in PBS (Invitrogen).

## Supporting Information

Movie S1 **Imaging islets in the normal pancreas.** 3D rendition of a normal islet as visualized *in vivo* by two-photon imaging. Note the coherent beta cell architecture and uniform GFP expression. Gridlines are 50 µm apart. For xyz dimensions see figure legend 1C.
(AVI)

Movie S2 **Imaging blood flow in the normal pancreas ascertains physiologic conditions.** In order to ascertain that local vascular supply is fully maintained, pancreatic islets (green) and their surrounding microvessels (red) were captured at high frame rates (insets represent single z-planes at 9fps) in normal animals. Blood flow and vascular integrity are shown here in 2 distinct pancreatic regions (Region 1/2 are MIPs from 43 and 35 planes respectively, each at 3 µm intervals) from a single representative animal. White scale bars are 100 µm. Pixel w/h for overview images is 1.5 µm, for insets 1 µm.
(AVI)

Movie S3 **Imaging of Diabetogenic Immunity in a Peptide-Induced Transfer Model.** Inflamed islets (green) are imaged at several stages of beta cell decay with varying levels of diabetogenic infiltrate (red). All movies were acquired at day 7–8 after cell transfer, right before clinical diabetes onset. For xyz dimensions, see figure legend 3. Time resolution is 1 minute.
(AVI)

Movie S4 **Imaging of leukocyte-beta cell interactions in a peptide-induced transfer model.** Isosurface rendering of leukocytes (red) interacting with beta cells (green), derived from the raw fluorescence data presented in Movie S3. Bars in lower left corner are 10 µm. Time resolution is 1 minute.
(AVI)

Movie S5 **Dynamics of pancreatic dendritic cells under steady-state conditions.** Dendritic cells (yellow) can be seen in the normal pancreas at relatively low densities, in no particular association with the islets (*left panel*). Grid lines are 50 µm apart. Injection of Texas-Red labeled dextran (magenta) reveals the dense vascular network in the pancreas. The dendritic cells constantly engage in interactions with the local microvasculature (*right panels*). For xyz dimensions, see figure legend 4. Time resolution is 1 minute.
(AVI)

Movie S6 **Imaging of pancreatic dendritic cells during diabetes development.** Visualization of dendritic cell (green, left panel; yellow, middle and right panel) recruitment to inflamed islets (*left panel*, white arrow). Once the DC enter the islet site, they virtually arrest and remain wedged in and around the islet for prolonged periods of time (*middle-right panel*). Real time injection of Texas-Red dextran (red) was performed to reveal the islet vasculature. Grid lines are 50 µm apart. All movies are 1 minute interval sequences containing MIPs of 30 planes spaced 6 µm apart and with pixel w/h of 1.5 µm.
(AVI)
